# A RECOMMENDATION FOR A UNIFORM DEFINITION OF DISTANCE CAREGIVING: A SCOPING REVIEW

**DOI:** 10.1093/geroni/igad104.3148

**Published:** 2023-12-21

**Authors:** Andrea Budnick, Farina Bünning, Adelheid Kuhlmey

**Affiliations:** Charité - Universitätsmedizin Berlin, Berlin, Berlin, Germany; Charité - Universitätsmedizin Berlin, Berlin, Berlin, Germany; Charité - Universitätsmedizin Berlin, Berlin, Berlin, Germany

## Abstract

Distance caregiving has increasingly attracted the attention of the gerontological research community. This indicates the importance of this caregiving arrangement in the face of progressive demographic change including fewer and fewer individuals providing local care. There have been attempts to define distance caregiving for more than two decades. The literature focuses predominantly on North America and Great Britain (e.g. Cagle & Munn, 2012; Franke et al., 2019). Although Cagle and Munn defined distance caregiving broadly, recent literature continues to focus on individual aspects, such as distance or time. Thus, future research on distance caregiving may benefit from using a uniform definition to compare findings across studies. Accordingly, we reviewed the literature of the last five years by means of a scoping review in 13 English and German databases. Following the systematic review on distance caregiving by Franke et al., we used May 2019 as the starting point of our search. Two independent raters classified the articles using prespecified inclusion and exclusion criteria. We identified a total of N=14,546 articles. After removal of duplicates, and title and abstract screening, we identified a total of n=59 articles for full text screening. We found the broad definition of distance caregiving by Cagle and Munn in 26 articles. The remaining articles referred to aspects like distance in miles or kilometers, time required, or other circumstances. In sum, our findings indicate that gerontological research on distance caregiving does not always build on existing knowledge.

